# An ecological study on childhood autism

**DOI:** 10.1186/1476-072X-11-44

**Published:** 2012-10-11

**Authors:** Sophie St-Hilaire, Victor O Ezike, Henrik Stryhn, Michael A Thomas

**Affiliations:** 1 Department of Health Management, Atlantic Veterinary College, University of Prince Edward Island, 550 University Avenue, Charlottetown, PE C1A 4P3, Canada; 2 Department of Biological Sciences, Idaho State University, 921 S. 8th Ave, Pocatello, Idaho, 83209, USA

**Keywords:** Idiopathic autism, Environmental factors, Drinking water, Air pollutants, Precipitation

## Abstract

**Background and methods:**

Idiopathic autism, suspected to be caused by exposure of genetically susceptible individuals to unknown environmental triggers, has increased dramatically in the past 25 years. The objectives of our study were to determine, using a linear regression model, whether the county prevalence of autism in the Pacific Northwest of the United States was associated with the source of drinking water for that county and whether this relationship was dependent on the level of environmental pollutants and meteorological factors in the county.

**Results:**

We found the previously reported relationship between precipitation and autism in a county was dependent on the amount of drinking water derived from surface sources in the county. We also found a positive association between the EPA’s risk of neurological disease and autism, but this relationship was only present in warm areas.

**Conclusions:**

Our study provides evidence for the hypothesis that environmental factors are associated with autism and that meteorological factors play a role in this relationship.

## Background

Autism spectrum disorders (ASDs) are a group of developmental disabilities characterized by impaired social skills and communication deficits, typically diagnosed by the age of three [1-3]. The incidence of ASDs has increased dramatically in the past 25 years, from 0.05% in the early 1980’s to 1.1% in 2008 [4-6]. The recent steady increase in ASDs, without a compensatory decrease in the diagnosis of other psychological conditions [7], is suggestive of an environmental disease caused by exposure to a risk factor(s) that is relatively widespread.

Despite its increasing incidence, the causes of the majority of cases of ASDs remain unknown. One of the difficulties in investigating these diseases is the use of broad case definitions. Further, symptoms vary between patients; however, it is fairly well accepted that ASDs develop prenatally or within the first few years of life and, at least with high functioning autism, occur more frequently in males than females [5,8].

Several researchers have hypothesized about the possible role of serotonin and gamma-aminobutyric acid (GABA) in autism. Whitaker-Azmitia [9] suggests that early exposure to high levels of serotonin while the brain is developing results in a negative feedback on the development of serotonin terminals, and studies have demonstrated this phenomenon in laboratory rats [10,11]. The reduction of serotonin terminals may then have cascading effects on a number of neurological pathways that are regulated by this neurotransmitter [12]. Other researchers have observed a dysfunction in the GABAergic system of autistic individuals [13]. In laboratory studies on neonatal rats, exposure to high levels of extracellular GABA-induced neuronal cell death which persisted over time [14].

Exposure to compounds that could result in hyperserotonemia or other imbalances in neurotransmitters in pregnant women or in infants less than 2 years of age, when the blood brain barrier is still permeable [9], could occur via a number of ways, including inhalation of chemicals, consumption of contaminated food and water, and exposure to prescription or other drugs. Fetal exposure to cocaine, which results in hyperserotonemia, has been associated with an increased risk of autism [15]. The administration of selective serotonin re-uptake inhibitors (SSRIs), or serotonin–norepinephrine reuptake inhibitors, in pregnant women is still practiced [16] and could result in higher than expected levels of serotonin in the fetus.

Psychiatric pharmaceuticals, such as SSRIs and GABA re-uptake inhibitors have been increasing in use since the late 1980’s and early 1990’s [17] and have been found in low levels in surface water and drinking water from surface sources [18-21]. Although the levels of pharmaceuticals in surface waters are below the predicted “no-effect concentrations” for adults and older children, and are considered to have “no appreciable” human health risk [22], it is unknown if, at these levels, there are effects on the developing brain. Further, these compounds are often found together and may behave synergistically.

Brain damage during development may also be a sequel of exposure to elevated levels of estradiol or estrogen-mimicking compounds [23]. In the last two decades, researchers have identified several areas of the brain responsible for mood, cognition, dementia, motor coordination, and excitability, where estradiol plays a role in regulating the release, transport, and uptake of different neurotransmitters, including serotonin and GABA [14,23-26]. There are numerous studies on the effects of endocrine disruptors on the serotonergic, dopaminergic, and noradrenergic systems of aquatic animals and mammals [27-30]. In Rhesus Macaques, estrogen has been shown to induce tryptophan hydroxylase gene expression [24], potentially resulting in altered neurotransmitter levels.

The effects of estradiol on the developing brain are not well understood. It is clear that exposure to endogenously derived estradiol is required for sexual differentiation; however, exposure to excessive levels of this hormone could result in permanent damage to the brain [23]. It has also been proposed that males may be at greater risk of embryonic exposure to high levels of estradiol due to the existing local production of this hormone from the conversion of testosterone within the brain [31], which could explain the higher rate of autism in males than in females [5].In a study on neonatal rats, the negative effect of high levels of GABA on the developing brain were exacerbated by pretreatment with estradiol, and this effect was more pronounced in males than females [14].

Estrogen-mimicking compounds have been found in the environment and in surface and drinking water in many areas around the world [32,33]. Given current theories on autism and the finding of low-level contamination of drinking water in the U.S., specifically drinking water from surface sources with pharmaceuticals including estrogenic compounds and other pollutants, we questioned whether drinking water might be a common source of exposure for numerous potential risk factors for ASDs. The objective of our study was to determine whether the prevalence of autism in a county was correlated with the source of drinking water for that county and whether this relationship was dependent on the level of environmental pollution in the county.

## Methods

Secondary data on county level autism prevalence were obtained from Waldman et al. [34]. The prevalence of autism was calculated for children aged 6 to 18 years of age in 2005 by county of residence from state agency data in Oregon and Washington and from the Department of Developmental Services in California. The denominator used in the prevalence calculation was the total number of school-aged children in each county reported by the 2000 U.S. Census Bureau. All races and both genders were included in the prevalence calculation. The counties with an autism prevalence of 0 and a base population of less than 1,000 were excluded from our analysis because the estimates were deemed unstable. Two counties were excluded using this criterion and, in total, 120 counties were included in the analyses. There was one outlier in the dataset and the analysis was run with and without this data point for comparison.

Data on the percentage of drinking water derived from surface water sources for each county were obtained from city-data.com [35]^a^. The county level risk of neurological diseases from toxic air emissions, derived from the National-Scale Air Toxics Assessment Program, was obtained from the U.S. Environmental Protection Agency (EPA) [36]. The county population density (number of individuals in a county per square mile) in 2000 and the acres of land used to grow crops in 1997 were obtained from the U.S. Census Bureau [37]. The number of acres of land used to grow crops was used to calculate the proportion of the county used as agricultural land (square mile of crops per square mile of the county), which was used as a proxy for pesticide use. The annual county level unemployment rate in 1990, 1995, and 2000 were averaged and included in our model as a measure of socioeconomic status. The average annual population suicide rate (per 100,000) in counties, between 2000 and 2004, was obtained from the Centers for Disease Control and Prevention [38] as a proxy for the rate of depression.

The 18-year average annual short wave radiation, heating degree-days (annual sum of degrees Celsius required to attain 18.3°C when the air temperature is less than 18.3°C), and average annual precipitation were downloaded from Daymet [39]. The spatial reference for the meteorological data was defined in ArcGIS (v. 9.3.1) using a projection file created by the Utah State University Spatial Data Group [40]. Data were then projected again to permit for the computation of the means by county using zonal statistics. Average county elevation was obtained from the National Oceanic and Atmospheric Administration’s (NOAA) National Climatic Data Center (NCDC) [41].

All explanatory variables were standardized to facilitate model building and the comparison of the coefficient estimates. A univariate regression analysis was conducted on each variable to determine which variables to consider in the multivariate analysis. All variables with a *p*-value greater than 0.50 were excluded from the remainder of the regression models. A multiple linear regression model was used to assess whether the prevalence of autism in a county was correlated to measures of local pollution (i.e. urban pollution, the EPA’s neurological disease risk from air emissions, and crop density), the annual county suicide rate, the percentage of drinking water derived from surface water sources, and meteorological parameters, controlling for the effects of unemployment rate (a measure of the socioeconomic status), and state where the autism diagnosis was made. Meteorological parameters, such as heating degree-days (HDD) and average annual precipitation, were included in the initial model because of their potential effects on pollution deposition, volatilization, and degradation [42-44]. Radiation was excluded because it was highly correlated with both HDD (r = −0.71) and average annual precipitation (r = −0.60).

A Box-Cox power transformation was performed with the model that included all potential main effects to determine whether the data required transformation. We added 0.01% to the two 0 values of autism in the dataset to allow this analysis. The percent autism in each county was subsequently transformed with a square root function to improve the fit of the linear regression model. Biologically plausible two-way interaction terms, such as percentage of drinking water derived from surface sources with precipitation, the EPA’s neurological disease risk from air emissions with precipitation and with HDD, and crop density and population density with precipitation, were included to assess whether these variables modified each other’s effects. When interaction terms were included, the primary variables were forced into the model regardless of their *p-*values. All other variables with *p*-values greater than 0.05 were systematically excluded from subsequent models by backward elimination until the final model included only statistically significant variables (*p*-value < 0.05). All statistical analyses were conducted in Minitab (Minitab 16^TM^, State College, PA, USA).

Interaction plots were made for all significant interaction terms by using the mean values for the variables (Table 1) in the (unstandardized) regression equation and changing the values of the main effect variables in the interaction terms to reflect the mean +/− one standard deviation. The square root of the proportion of autism produced by the model was back transformed (Y^2^) before it was graphed.

**Table 1:** Descriptive statistics for variables included in our regression analysis and results of the univariate regression analysis for each variable

**Variable**	**Mean**	**Standard deviation**	**25% quartile**	**75% quartile**	**Regression Coefficient**	**t- statistic**	***p*-value**
**State Name**							
% autism in Washington counties (n=35)	0.24	0.13				Reference state	
% autism in California counties (n=57)	0.18	0.07			−0.149	−9.32	<0.001
% autism in Oregon counties (n=28)	0.68	0.25			0.291	13.17	<0.001
Drinking water derived from surface sources (%)	48.51	34.70	15.0	79.75	0.073	4.09	<0.001
Average unemployment (%)	7.43	3.03	5.21	8.96	−0.025	−4.28	<0.001
Suicide rate (per 100,000)	14.24	5.09	10.13	17.10	0.074	4.17	<0.001
**Pollutants**							
EPA risk of neurological disease from air emissions (hazard index )	0.059	0.054	0.032	0.061	0.071	3.95	<0.001
Crop density (Square km of land used to grow crops per county square km )	0.15	0.18	0.0247	0.21	−0.019	−0.97	0.334
Population (number of people per square km)	361	1563	19	163	−0.008	−0.41	0.685
**Meteorological parameters**							
Average annual precipitation (cm)	108.87	70.97	54.97	165.71	0.092	5.37	<0.001
Median elevation (ft)	440.3	479.1	146.0	516.8	−0.008	−0.42	0.676
Heating degree days (^0^C*days)	2933.3	1045.7	1914.0	3718.4	0.030	1.58	0.118

The outcome variable used in the analyses was the square root transformed percent of autism for each county and the regression coefficients are for standardized variables. Regressions were run using data from 120 counties in Washington, Oregon, and California.

## Results

**Figure 1:**
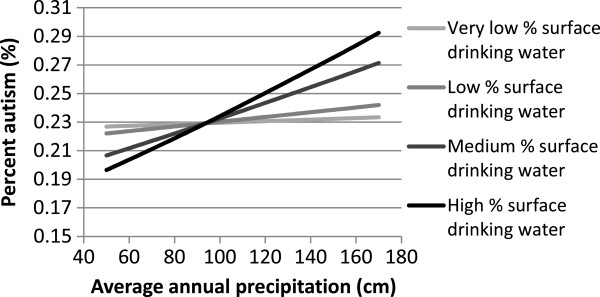
**Predicted effect of precipitation on county prevalence of autism in 2005 when different levels of drinking water were derived from surface sources (very low = 3.3%, low = 15% , median =53.5 %, and high = 79.8%) using our final model (unstandardized) and maintaining all other other variables in the model constant at their mean value.** Expected percent autism ^0.5^ = 0.47529 - 0.000927187 *Surface drinking Water - 0.00867663 *Avg. unemployment + 0.0000236374* annual precipitation −0.0000230421* HDD + 2.53316 * EPA Neurological Disease Risk+ 0.00733637 *suicide rate + 0.00000989236 * precipitation* drinking water −0.00069674* EPA Neurological Disease Risk * HDD. The reference state (Washington), was used to create the interaction plot and data were back transformed before they were graphed.

**Figure 2:**
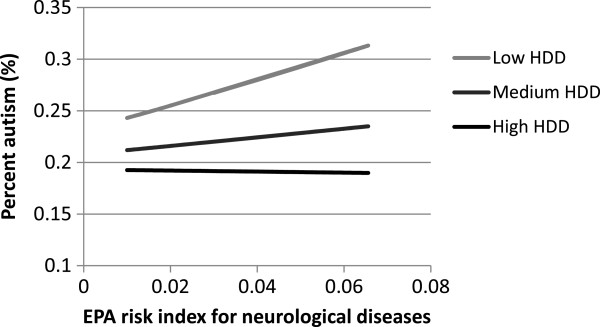
**Predicted effect of EPA’s risk index for neurological diseases based on air emmissions on county prevalence of autism in 2005 for different levels of heating degree days (low =1914, median =3004, and high = 3718) using our final model (unstandardized) and maintaining all other other variables in the model constant at their mean value.** Expected percent autism ^0.5^ = 0.47529 - 0.000927187 *Surface drinking Water - 0.00867663 *Avg. unemployment + 0.0000236374* annual precipitation −0.0000230421* HDD + 2.53316 * EPA Neurological Disease Risk+ 0.00733637 *suicide rate 0.00000989236 * precipitation* drinking water −0.00069674* EPA Neurological Disease Risk * HDD. The reference state (Washington), was used to create the interaction plot and data were back transformed before they were graphed.

The range of autism rates in our study was 0 to 1.26% (mean 0.31%). The assumptions of homoscedasticity and normal distribution were met after we transformed the proportion of autism by the square root function. Median elevation was excluded from our initial multiple regression analysis based on its high *p* values in the univariate analysis (Table 1). All other variables were initially included in our model. Our final model, which included 7 variables and 2 interaction terms, explained approximately 72% of the variation in autism between counties (R^2^ = 0.724 and R^2^ predicted =0.685). We found the positive association between precipitation and autism reported by Waldman et al. [34] was dependent on the county’s source of drinking water (Table 2; Figure 1). Based on our model, the relationship between precipitation and autism was stronger in counties that derived a large proportion of their drinking water from surface sources than in counties that derived very little water from surface sources (Figure 1). Other significant variables associated with county level autism included state of residence, average annual suicide rate in the county, unemployment rate, risk of neurological diseases from toxic air emissions, and average annual heating degree days (Table 2). Autism was positively correlated to suicide rate and negatively correlated to the unemployment rate in a county (Table 2). The EPA’s neurological disease risk score (toxic air emissions) was positively correlated with the proportion of autism in counties with low HDD (warmer counties), but this relationship was not present when HDD was high (Figure 2). The other two pollution indices (crop and population density), were not statistically significant in our models, nor was the interaction between precipitation and crop density (*p* >0.05), or EPA air emissions and precipitation (*p* = 0.10).

**Table 2:** Final general linear model showing significant predictors of autism and interaction terms (the analysis include the outlier county from Oregon)

**Term**	**Coefficient**	**T statistic**	***p*-value**
Constant	0.546	46.12	<0.001
State of Washington (Reference State)			
California	−0.155	−7.53	<0.001
Oregon	0.215	10.63	<0.001
% Surface drinking water	0.005	0.45	0.654
Annual Suicide rate	0.035	2.71	0.008
Average annual unemployment rate (1990,1995, and 2000)	−0.026	−2.37	0.020
Annual Precipitation	0.035	2.71	0.008
EPA Neurological Disease Risk	0.026	2.28	0.025
Heating Degree Days (HDD)	−0.067	−4.35	<0.001
EPA Neurological Disease Risk X HDD (interaction)	−0.040	−2.19	0.031
Precipitation X % Surface drinking water (interaction)	0.025	2.13	0.035

The adjusted R^2^ for the model was 0.724. We used standardized explanatory variables in our model to enable comparison of coefficients.

Approximately 30% of the variation in the data could be explained by the state where the diagnosis was made (R^2^_model with state_= 0.724 vs. R^2^_model without state_ = 0.440). On average, California had lower autism rates than Washington, but Oregon had higher rates than Washington both overall and when adjusting for other factors (Tables 1 and 2).

## Discussion

The effects of pollutants on the health of animals and humans is well described in the literature, and the roles of temperature, precipitation, and other meteorological parameters on pollution exposure levels are also well known [42-47]. Despite this knowledge, meteorological variables are relatively infrequently considered in environmental disease models, even when studies are conducted over large spatial scales. Omitting these variables may lead to bias due to confounding or interactions between pollutants and environmental parameters. Several years ago, Waldman et al. [34] published that the prevalence of autism in the U.S. Pacific Northwest was correlated to the amount of annual precipitation in the county. We used the autism data from Waldman et al. [34] to further explore the relationship between the environment, pollutants, and autism. By including several measures of local pollution, and a possible route of exposure for pollutants in our regression model we provide a plausible explanation for the association between precipitation and autism reported by Waldman et al. [34]. Our study suggests precipitation was more strongly associated with occurrence of autism in counties where the drinking water was primarily derived from surface sources (Figure 1). In fact our model suggests the relationship between precipitation and autism is greatly reduced when countries do not derive their drinking water from surface sources (Figure 1). This suggests the relationship between precipitation and autism may be linked to drinking water. There are a number of biologically plausible explanations for the interaction between precipitation and drinking water source that should be investigated.

There have been numerous studies on the deposition of semi-volatile organic pollutants and heavy metals in rain and snow [43,46,47] that establish the biological plausibility that areas of heavy precipitation may also have higher levels of contaminants in their surface water. The fact that the association between autism and precipitation was partially dependent on the amount of drinking water derived from surface sources suggests a possible route of exposure for environmental contaminants. Although direct deposition of pollutants by precipitation is one way that surface water may be contaminated, it is also possible that precipitation increases run-off from agricultural, industrial, and urban sources in local surface waters [43]. Further, precipitation may also result in the agitation of contaminated sediments in surface waters, resulting in the disassociation and re-suspension of pollutants into the water column. In some areas around wastewater treatment plants the levels of some pollutants, including estrogenic compounds and heavy metals, can be higher in sediment than in the water column [32,48], so agitation of these sediments could increase the release of pollutants.

We attempted to evaluate whether agricultural activity was associated with the proportion of autism in a county by including the variable crop density in our model, but it was not statistically significant (Table 1). We also evaluated whether or not the effect of agriculture was dependent on the level of precipitation (i.e. via run-off) by including an interaction term, but it was also not significant. Further, urban pollution was also not significantly associated with autism in our study (Table 1), but it is possible that by including variables such as drinking water source and air emissions, which are sometimes correlated to population density, we explained some of the variation in autism that would have been attributed to rural or urban areas.

We did observe a positive association between the EPA’s risk of neurological disease, which was based on 23 air emission parameters, and autism in counties with low HDD (or the warmer counties in our study) (Table 2 and Figure 2). Air temperature affects the partitioning of air pollutants between solid and gaseous states [42,43], therefore it is possible that exposure to air emissions varies in direct relation to air temperature. Heating degree-days was used to represent temperature because it provides an estimate of the duration or extent of cold weather in an area (annual sum of degrees Celsius required to attain 18.3°C when the air temperature is less than 18.3°C). In relatively warm counties (i.e. lower HDD), we detected a positive association between the EPA’s risk of neurological disease from air emissions and the prevalence of autism (Figure 2). A similar positive association between exposure to heavy metal and chlorinated solvents air emissions and autism was previously reported for a smaller geographical area in California and in Texas [49,50]. Interestingly, this relationship was consistent across the Pacific Northwest only in areas without extreme cold temperatures. The significant interaction term in our model provides a possible explanation for the occasional conflicting results reported in different studies on air emissions and autism [51], and suggests temperature may affect the relationship between air emissions and disease.

Our study was also consistent with other observational studies with regard to the association between autism and socioeconomic status. We found a negative association between unemployment rate and autism, which is consistent with other recent studies that have found the prevalence of autism is higher in families that have a higher socioeconomic status (or lower unemployment) [52]. Durkin et al. [52] suggest this may reflect access to services.

The state of residence was significantly associated with the county level prevalence of autism, and explained a large proportion of the variance. In fact, almost half of the explained variation in autism prevalence in this study was found to be attributable to the state where the county was located (R^2^ = 0.724 vs. R^2^ = 0.440). The state of residence where the diagnosis was made may capture many different factors that exist among the different states including access to services, but given the subjective nature of the diagnosis of autism, it likely reflects the differences in the diagnostic and classification criteria. To reduce the variation in the diagnostic criteria, it is recommended that, in the future, a standard definition and system of classification be used to assure consistency in the identification of individuals with autism. The Autism and Developmental Disorders Monitoring (ADDM) network has a system to measure, compare and contrast, and monitor autism rates across selected areas in the U.S. using well defined standards, but county level ADDM data are limited to 762 counties (out of over 3000) [53] and are not publicly available.

Testing our model with a larger dataset such as that of the ADDM would also permit for the evaluation of more complicated interactions between possible sources of contaminants and potential modifiers of these contaminants and routes of exposure. Given the biological plausibility for these intricate relationships (i.e. drinking water source by precipitation by source of pollution), and the preliminary findings from this study that suggests basic interactions exist (i.e. precipitation and drinking water as well as air emissions and HDD), it is important that future studies test these hypotheses.

Another biologically plausible explanation for the relationship between the precipitation/drinking water source interaction term and county level autism that should be further explored is that rain may be correlated to depression which may be correlated to the usage of psychotropic pharmaceuticals in a county. We were not able to confirm this relationship because data on use of these pharmaceuticals were not available to us at the county level. However, a strong positive association was detected between a county’s suicide rate and its autism rate, after controlling for possible confounders such as the county’s unemployment rate (Table 2). Further, when this variable was excluded from the model (data not shown) the model’s coefficient for precipitation increased, which suggests that suicide rate may explain some of the variation previously attributed to precipitation. A link between depression and autism would be consistent with the hyperserotonin hypothesis proposed by Whitaker-Azmitia [9] and other recent studies involving model organisms [54].

Other limitations of the study, besides the small sample size and the crude approaches used for some measures of pollution, were those that are common to most ecological types of observational studies. These include the inability to control for all possible confounding variables and the potential for lag time bias that may have resulted in misclassification of the exposure status because it occurred several years before the children were diagnosed with autism. Lastly, it could not be concluded that the association between the percentage of drinking water derived from surface water sources and the prevalence of autism at county level translates to the individuals (the actual exposures of autistic and non-autistic children in our study were unknown). Therefore, while the associations detected can only be applied to the county level, these results suggest possible areas where further research should be conducted to establish whether the risk factors identified at the county level extend to the individual.

## Conclusions

Although the results of this epidemiological study are limited to correlations at the group level due to the type of study, they are suggestive of an environmental trigger for autism, correlated to precipitation but only significant in counties that derive their drinking water from surface sources. Further our findings corroborate other studies that have found specific types of air emissions are positively correlated with autism, at least in areas with warmer weather. This study helps explain the findings from the earlier analyses by Waldman et al. [34]; however, because we analyzed the same autism dataset as this research group the findings should be confirmed with additional data from other areas in the U.S. and using an observational study design that permits testing of specific hypotheses. Given the recent findings by different research groups that link perinatal alterations in neurotransmitters and receptor development with autism, the presence of compounds in surface and drinking water that could biologically cause these neurological changes, and the widespread increasing pattern of autism reported globally in industrialized countries consistent with a widespread exposure to one or more risk factors, we feel it is important that the findings from this study be further investigated to clarify whether or not drinking water source and local sources of pollution play a role in autism.

## Endnotes

^a^To obtain data on different counties the name of each county and state has to be manually changed in the website address.

## Abbreviations

ADDM: Autism and Developmental Disorders Monitoring; ASDs: Autism spectrum disorders; SSRIs: Selective serotonin re-uptake inhibitors; GABA: Gamma-aminobutyric acid; CDC: Centers for Disease Control and Prevention; EPA: Environmental Protection Agency; HDD: Heating degree days which is the annual sum of degrees Celsius required to attain 18.3°C when the air temperature is less than 18.3°C; NCDC: National Climatic Data Center; NOAA: National Oceanic and Atmospheric Administration.

## Competing interests

The authors declare that they have no competing interests.

## Authors’ contributions

MT provided the idea for the project. SS and VE developed the hypothesis, designed the study, and interpreted the data. VE also extracted the data on water source for individual counties. SS and HS conducted the statistical analysis. SS wrote the manuscript. All authors participated in the review and final approval of the manuscript.
